# Implication of Hemodynamic Assessment during Durable Left Ventricular Assist Device Support

**DOI:** 10.3390/medicina56080413

**Published:** 2020-08-15

**Authors:** Teruhiko Imamura, Nikhil Narang

**Affiliations:** 1 Second Department of Internal Medicine, University of Toyama, 2630 Sugitani, Toyama 930-0194, Japan; 2 Advocate Christ Medical Center, Oak Lawn, IL 60453, USA; nikhil.narang@gmail.com

**Keywords:** right heart catheterization, ventricular assist device, congestion

## Abstract

Durable left ventricular assist device therapy has improved survival in patients with advanced heart failure refractory to conventional medical therapy, although the readmission rates due to device-related comorbidities remain high. Left ventricular assist devices are designed to support a failing left ventricle through relief of congestion and improvement of cardiac output. However, many patients still have abnormal hemodynamics even though they may appear to be clinically stable. Furthermore, such abnormal hemodynamics are associated with an increased risk of future adverse events including recurrent heart failure, gastrointestinal bleeding, stroke, and pump thrombosis. Correction of residual hemodynamic derangements post-implantation may be a target in improving longitudinal clinical outcomes during left ventricular assist device support. Automatic and timely device speed adjustments considering a patients’ hemodynamic status (i.e., with a smart pump) are potential improvements in forthcoming devices.

## 1. Introduction

Despite considerable improvement in available heart failure-specific medical therapies including beta-blockers, angiotensin-converting enzyme inhibitors, angiotensin II receptor blockers, aldosterone antagonists, angiotensin receptor-neprilysin inhibitors, and arginine vasopressin type II receptor antagonists, morbidity and mortality in patients with advanced heart failure remain exceedingly high [1].

In addition to mechanical circulatory support technologies including the intra-aortic balloon pump, extra-corporeal membrane oxygenation, and percutaneous axial-flow left ventricular assist device (LVAD), cardiac replacement therapy (heart transplantation and durable LVAD) remains the gold-standard therapy for those with refractory stage D heart failure [2]. Given the considerable shortage of donors’ hearts, durable LVAD therapy is a widely-used tool in bridging patients to eventual heart transplantation or as destination therapy in non-transplant candidates [3].

LVAD therapy improves survival in patients with advanced heart failure compared to medical therapies alone [4,5]; however, readmissions due to various device-related comorbidities remain unsatisfactorily high. In the MOMENTUM 3 trial (Multicenter Study of MagLev Technology in Patients Undergoing Mechanical Circulatory Support Therapy with HeartMate 3) [6,7], patients with advanced heart failure were randomly assigned to the novel HeartMate 3 LVAD (Abbott, Abbott Park, IL) arm and the prior generation HeartMate II LVADs (Abbott, Abbott Park, IL). Patients in the HeartMate 3 LVAD arm experienced significantly higher survival-free from stroke compared with the HeartMate II LVAD cohort. However, the incidence of several hemocompatibility-related adverse events including gastrointestinal bleeding remained comparably high despite novel pump technology [8,9].

LVADs correct hemodynamic derangements by mechanically unloading the failing left ventricle that decreases intra-cardiac pressure and subsequently increasing systemic circulation that increases total cardiac output. However, one report showed that many LVAD patients have abnormal hemodynamics despite appearing clinically stable in the ambulatory setting [10]. Furthermore, it has recently been demonstrated that such abnormal hemodynamics may be associated with various future adverse events during LVAD support [11]. In this review, we will discuss the association between invasive hemodynamics and adverse events, clinical strategies to address abnormal hemodynamics, and non-invasive hemodynamic monitoring.

## 2. Hemodynamics and Adverse Events

**Figure 1:**
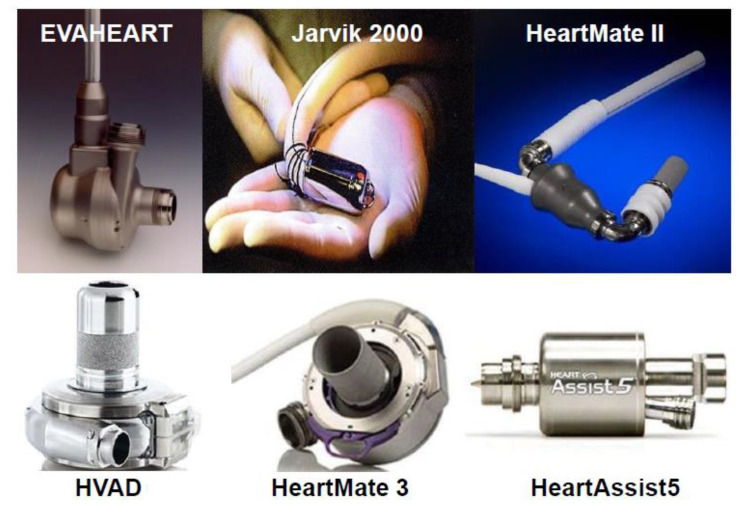
Currently available durable left ventricular assist devices (reused with approval [12]). HVAD: HartWare left ventricular assist device.

Durable LVAD technology is improving, from para-corporeal models to the implantable and smaller iterations, including both pulsatile and continuous-flow types. Notably, the dominant types in the current era are implantable continuous-flow devices (Figure 1) [12]. The underlying goal of durable LVAD therapy from the first prototypes is to unload the left ventricle, increase systemic circulation, and ameliorate pulmonary congestion.

Nevertheless, our group recently found that many LVAD patients had abnormal hemodynamics despite showing no apparent clinical symptomology [10]. Here, we defined abnormal hemodynamics as central venous pressure > 12 mmHg, pulmonary capillary wedge pressure > 18 mmHg, and cardiac index < 2.2 L/min/m^2^. Notably, we can measure directly or calculate various other hemodynamic parameters using right heart catheterization, as discussed later.

Particularly, many LVAD patients seem to have inappropriately elevated central venous pressure indicative of sub-clinical right heart failure [13,14]. LVADs are designed to reduce the right ventricular afterload by reducing pulmonary artery pressures, whereas the preload experienced by the right ventricle increases due to the improved systemic circulation. Considerable unloading of the left ventricle and reduction in left ventricular size also modifies the geometric relationship between left ventricle and right ventricle. The increased preload experienced by the unprepared right ventricle as a result of improved systemic circulation may worsen right ventricular function in some patients acutely and others over time [15]. As a result, central venous pressure can remain unchanged following LVAD implantation on average [10]. Notably, the degree of hemodynamic improvements can vary greatly as governed by device speed, existing patient-related factors, and device type.

For several reasons, we believe that invasive right heart catheterization should be routinely performed following LVAD implantation [16]. First, many patients may have residual abnormal hemodynamics following LVAD support. Second, such abnormal hemodynamics are sometimes a challenge to clinically detect by physical examination alone [17]. Third, as discussed below, such abnormal hemodynamics are associated with future adverse events following LVAD implantation. Recently, several non-invasive procedures to estimate hemodynamic parameters have been studied and clinically implemented [12]. Such methods might prove to be clinically useful when right heart catheterization is not readily available [18].

### 2.1. Heart Failure

Our team recently demonstrated that the presence of abnormal hemodynamics post-LVAD implantation, even without heart failure symptoms, was associated with future instances of clinical volume overload and heart failure recurrence [13]. As one would expect, abnormal hemodynamics reflect insufficient mechanical unloading of the left ventricle, causing symptoms of dyspnea and volume retention due to pulmonary and/or systemic congestion. In addition to the device speed settings, other factors also affect the hemodynamic status of patients with LVADs. The clinical implications of heart failure-specific therapies are strongly validated as a disease-modifying agent in non-LVAD populations [1], whereas the effect in LVAD patients has not been robustly evaluated. Nevertheless, emerging data have suggested that guideline-directed medical therapies for heart failure may improve clinical outcomes also in LVAD patients [19]. This phenomenon may be at least partially explained by neurohormonal modulation leading to some degree of cardiac reverse remodeling and further improvement of intracardiac hemodynamics [19].

**Figure 2:**
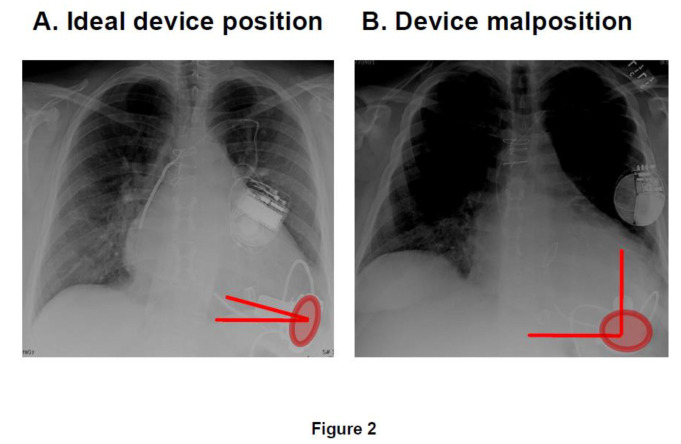
Recommended positioning of HeartWare left ventricular assist devices (reused with approval [21]) (**A**) ideal position; (**B**) device malposition. The horizontal angle of inflow cannula (angle between the two red bars) is narrow in the ideal position; whereas it is wide in the malposition. Also, the area of pump (red circle) is small in the ideal position; whereas it is large in the malposition.

Optimal device positioning also affects the device’s ability to effectively unload the left ventricle, with malposition of the inflow cannula associated with an increased risk of future heart failure exacerbations [20]. Lateral displacement of the inflow cannula in the HeartWare LVAD (Medtronic, Minneapolis, MN) might cause abnormal stasis and blood pooling in the left ventricle resulting in inefficient unloading (Figure 2) [21,22,23]. Further data are needed to better understand the relationship between implant technique and device positioning.

### 2.2. Gastrointestinal Bleeding

Bleeding, particularly gastrointestinal bleeding, is among the most common comorbidities during LVAD support with an estimated incidence of 25% [24]. Some gastrointestinal bleeding events are refractory to all available therapeutic strategies including octreotide, danazol, and thalidomide in addition to the termination of antiplatelet and anticoagulation therapies [25].

**Figure 3:**
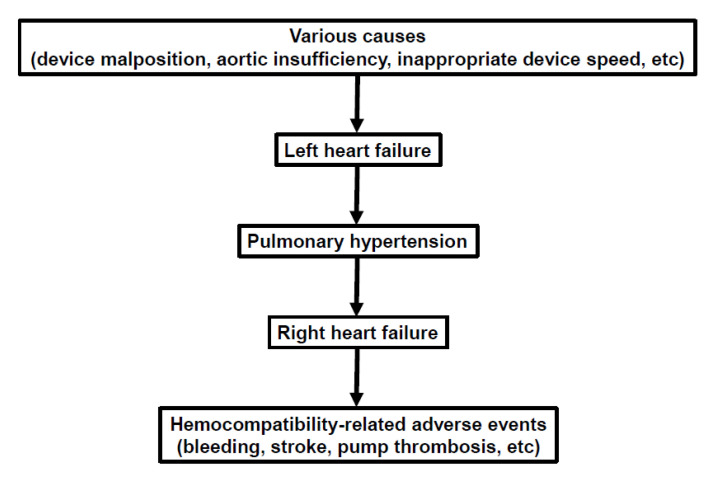
Hypothesized scheme of abnormal hemodynamic conditions and clinical outcomes.

The mechanism of gastrointestinal bleeding is multifactorial [26]. In addition to antiplatelet and anticoagulation therapies, along with acquired von Willebrand disease, inappropriate activation of inflammatory and angiogenesis cascades triggering rises in tumor necrosis factor-alpha and angiopoietin-2 may be the primary mechanism of abnormal arteriovenous malformations in the gastrointestinal tract. This might be the most common cause of LVAD-related gastrointestinal bleeding, as opposed to polyps or gastrointestinal mucosal ulceration [27,28]. It is plausible that abnormal hemodynamics, particularly systemic congestion due to elevated central venous pressures, are associated with the activation of these systems (Figure 3) [29]. Recently, therapies including omega-3 fatty acid and digoxin administration were found to be associated with fewer gastrointestinal bleeding events, possibly due to suppression of these maladaptive cascades [30,31,32]. Genetic and dietary factors might also have a considerable impact on angiogenesis activity, given a considerably lower observed incidence of gastrointestinal bleeding in Japanese cohorts [33]. Further molecular biological analyses are warranted to clarify the relationship between hemodynamics, the angiogenesis cascade, and gastrointestinal bleeding.

### 2.3. Stroke

The most apparent risk factor for LVAD-associated stroke is uncontrolled systemic blood pressure [34]. This relationship of blood pressure to incident risk of stroke was best defined in the ENDURANCE (A Clinical Trial to Evaluate the HeartWare Ventricular Assist System) Supplemental trial, where elevated mean blood pressure was associated with a higher incidence of stroke during HeartWare LVAD support [35]. Furthermore, abnormal hemodynamics, particularly right heart failure with elevated central venous pressure, may increase the risk of incident stroke (Figure 3) [13]. We postulate that an inflammatory cascade activates due to chronic and systemic congestion which may, in turn, increase cerebral vasculature vulnerability and the potential risk of stroke [29]. Another explanation may be a decrease in blood flow through the device because of impaired right ventricular function, contributing to increased stasis of flow and potentially increased risk of clot formation in the device [11].

### 2.4. Pump Thrombosis

Pump thrombosis is one of the major causes of device malfunction that requires device exchange [36]. Abnormal LVAD hemodynamics may also increase the risk of incident pump thrombosis (Figure 3) [11]. Similar to other events, the mechanism of pump thrombosis is multifactorial. Sub-therapeutic anticoagulation is the most common cause, though device malposition as defined by the narrow-angle between the inflow cannula and pump body in HeartMate II LVAD, is also associated with an increased risk of pump thrombosis [37]. With a similar pathophysiologic mechanism of clot formation which may increase stroke risk, reduced flow through the pump motor due to right heart failure may increase also increase the risk of pump thrombosis [11].

## 3. Hemodynamic Patterns by Disease Process

Several unique hemodynamic patterns vary by disease state including right heart failure, pulmonary hypertension, and aortic insufficiency.

### 3.1. Right Heart Failure

We should state at first that there is no comprehensive and consistently agreed-upon definition of right heart failure [38]. Right heart failure clinically may manifest simply as worsening systemic congestion, whereas right ventricular function is best evaluated using echocardiography through assessment of tricuspid annular plane excursion and right ventricular fractional area change.

As mentioned above, right heart failure remains a highly morbid complication of contemporary LVAD therapy. Despite the pump’s purpose to restore systemic perfusion in the failing heart by unloading the left ventricle, adverse right ventricular remodeling resulting from longstanding left ventricular failure is common and challenging to correct by durable mechanical circulatory support alone. Following LVAD implantation, right ventricular preload dramatically increases due to improved systemic circulation. The right ventricle, however, is often unprepared for this drastic increase in flow due to maladaptive structural changes from the afterload of a chronically failing left heart. As a result, right heart failure can become apparent both early and in later periods following LVAD implantation. Furthermore, a decrease in the size of the left ventricle due to mechanical unloading also facilitates a geometrical unbalance between the left and the right ventricle, resulting in further impairment of normal right ventricular contractile mechanics [15].

Elevated central venous pressures in the setting of normal pulmonary capillary wedge pressure are one of the hallmarks of right heart failure and tend to be difficult to correct alone through mechanical unloading [39]. Right heart failure is also associated with above-discussed hemocompatibility-related adverse events including bleeding and systemic thromboembolism (Figure 3) [11].

**Figure 4:**
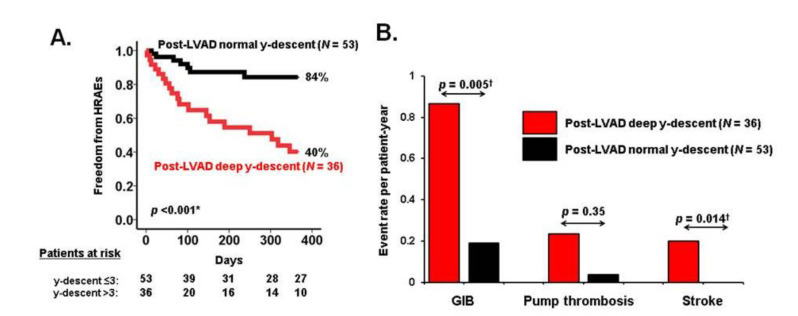
Impacts of y-descent on hemocompatibility-related adverse events during left ventricular assist device (LVAD) support (reused with permission [42]). * *p* <0.05 by log-rank test. ^†^ *p* <0.05 by Mann–Whitney U test. HRAE, hemocompatibility-related adverse events; GIB, gastrointestinal bleeding. A deep y-descent was defined as y-descent depth >3 mmHg from the mean right atrial pressure. A deep y-descent following LVAD implantation was associated with lower freedom from HRAEs, predominantly due to GIB and stroke.

Invasive right heart catheterization provides the clinician valuable information regarding right ventricular performance. Pulmonary artery pulsatility index, which is calculated as a pulse pressure of the pulmonary artery divided by the central venous pressure, is a recently proposed index of right ventricular function [40], with a cut-point of <1.85 associated with post-LVAD right ventricular failure [41]. Furthermore, we showed that a deep y-descent of the right atrial waveform obtained via right heart catheterization is another surrogate of right ventricular dysfunction, and is associated with echocardiography-derived right ventricular dysfunction along with poor clinical outcomes (Figure 4) [42].

### 3.2. Pulmonary Hypertension

Many patients with advanced heart failure have secondary pulmonary (combined pre and post-capillary) hypertension due to chronically elevated left-sided filling pressures [43]. However, many patients seemingly reverse their pre-existing pulmonary hypertension following LVAD implantation due to continuous mechanical unloading of the left ventricle [44,45]. Nevertheless, there are subgroups of patients that have residual pulmonary hypertension even following LVAD implantation, which is associated with worsening long-term right ventricular dysfunction [46]. Our group recently proposed an index to assess refractory pulmonary vasculature adverse remodeling. We described this as “decoupling”, which is defined as a gradient of >5 mmHg between diastolic pulmonary artery pressure and pulmonary capillary wedge pressure [47]. We observed that many patients have decoupling, irrespective of preoperative pulmonary hypertension. The presence of this gradient represents an afterload on the right ventricle that may contribute to progressively worsening right ventricular dysfunction. This ultimately leads to worsening congestion and an increased risk of hemocompatibility-related adverse events as discussed above (Figure 3) [48]. Of note, there is no general consensus about the best definition of pulmonary vasculopathy in post-capillary pulmonary hypertension, and several other indexes have been proposed, including pulmonary vascular resistance, trans-pulmonary artery pressure gradient, and pulmonary artery compliance [49]. Further studies are warranted to investigate the detailed association among them and their clinical impacts on the LVAD cohort.

### 3.3. Aortic Insufficiency

Aortic insufficiency is a unique and progressive comorbidity during long-term LVAD support. Continuous left ventricular unloading leads to aortic valve closure and pressure increases in the aortic root via the outflow graft. This can lead to valvular degeneration, and subsequent continuous and eccentric valvular regurgitation [50]. Aortic insufficiency increases left ventricular end-diastolic pressure. This consequently may increase right ventricular afterload (i.e., secondary pulmonary hypertension) leading to more strain on right ventricular function over time [51]. Aortic insufficiency, like many other LVAD-related complications, is associated with impaired functional capacity and increased mortality [50]. Notably, aortic insufficiency develops at similar historical rates even in patients with the most contemporary devices (HeartMate 3) [52].

The severity of aortic insufficiency is assessed usually using conventional color Doppler echocardiography for visual estimation. Accurate quantification is challenging given non-physiologic continuous and eccentric regurgitant flow. Our group recently proposed several methods to more accurately quantify the severity of aortic insufficiency [53].

First, we can estimate the degree of aortic insufficiency using a device flow monitor, which is equipped in the HeartWare LVAD [54]. This stems from the concept that elevated left ventricular diastolic pressure results in increased device flow during diastole. Severe aortic insufficiency equalizes the pressure between the aorta and left ventricle, minimizing the pressure gradient, resulting in increased pump flow. This allows for a non-Doppler modality to estimate aortic insufficiency.

**Figure 5:**
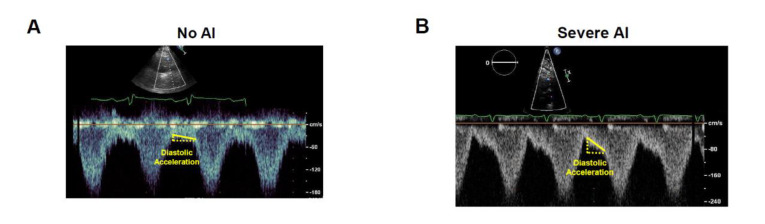
Pulse-Doppler echocardiography obtained at the outflow graft of left ventricular assist device (reused with permission [55]).The regurgitant fraction is estimated using the slope of diastolic acceleration (yellow bar). (**A**) no aortic insufficiency; (**B**) severe aortic insufficiency.

Second, we can quantify the severity of aortic insufficiency using Doppler echocardiography obtained at outflow graft (Figure 5) [53]. The concept is similar to the above-described flow monitor, with the regurgitant fraction estimated from the slope of blood flow. Interestingly, the severity of aortic insufficiency is generally greater when we use these novel methods compared to conventional visual estimation. Using these novel methods, many LVAD patients may have significant aortic insufficiency before clinical symptomology develops. Generally, the only definitive therapies are valve replacement or urgent heart transplantation [55].

## 4. Hemodynamic-Guided Optimization

As discussed above, abnormal hemodynamics are associated with various adverse clinical outcomes during LVAD support. Furthermore, there are several hemodynamically unique comorbidities during LVAD support which have unique and specific management strategies.

Our group has recently proposed a hemodynamic ramp test, in which hemodynamics are measured using right heart catheterization at each LVAD speed interval [10]. There are two major purposes of this test. First, we can understand the hemodynamic status at each speed interval from which we can determine the need for medication adjustments. Second, we can adjust the device speed to better optimize the patient’s hemodynamic profile with a primary target of right atrial pressure < 12 mmHg, pulmonary capillary wedge pressure < 18 mmHg, and cardiac index > 2.2 L/min/m^2^. We simultaneously perform echocardiography to assess for aortic valve opening, to understand the interventricular septum position, and to determine the presence of mitral valve regurgitation at each speed interval [39]. The Ramp-it-up trial demonstrated the prognostic implications of this specific test in a prospective randomized control setting [16].

New therapeutic targets for other LVAD-related comorbidities are being actively investigated. Data regarding therapies for LVAD-associated right heart failure including oral inotropes or newly developed vasopressin type-2 receptor antagonists are emerging [56]. Pulmonary hypertension might be improved by pulmonary hypertension specific therapies or device speed adjustment [47,49]. Some patients, however, may continue to have persistently elevated pulmonary artery pressures despite a normalized pulmonary capillary wedge pressure and despite attempts at speed optimization [57]. For patients with severe aortic insufficiency, device speed adjustments are generally not durable as a corrective measure [58]. Transcatheter aortic valve replacement is emerging as a potential salvage therapy in severely symptomatic patients who are not optimal surgical candidates for valve replacement or heart transplantation [59].

## 5. Non-Invasive Assessment of Hemodynamics

The static nature of right heart catheterization is the principal limitation of using hemodynamics in informing hourly and daily decision making for the majority of patients with chronic heart failure. Notably, right heart catheterization testing during exercise might partially overcome such limitations [60]. A patient’s hemodynamic status is time-dependent and can change dramatically during a hospitalization. Ideally, hemodynamics should be measured repeatedly or continuously [12]. However, routine invasive assessments in LVAD patients carry a procedural risk given the need for continuous anticoagulation. Herein, non-invasive hemodynamic assessment methods are needed to better guide clinical management of the LVAD patient.

### 5.1. CardioMEMS

**Figure 6:**
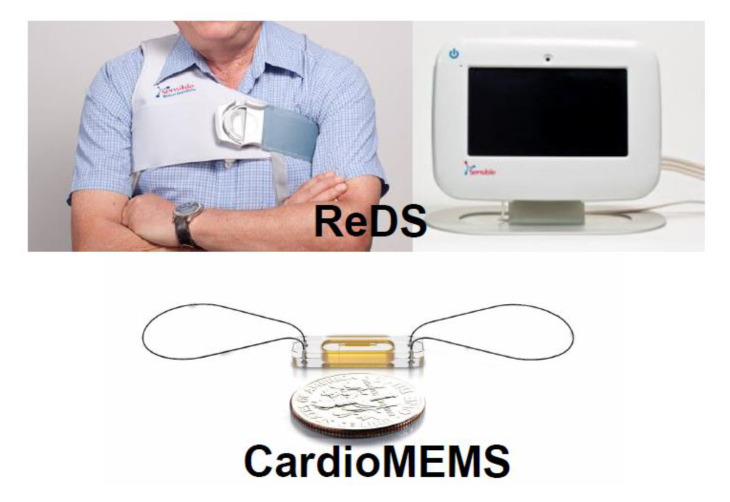
Examples of novel non-invasive technologies to measure hemodynamic parameters (reused with permissioin [12]). ReDS, remote dielectric sensing.

CardioMEMS was originally developed to monitor pulmonary artery pressures in patients with chronic heart failure using a sensor percutaneously implanted in the main pulmonary artery (Figure 6) [61]. Its use is increasing in routine heart failure care, given the reduced risk of heart failure hospitalizations in patients with CardioMEMS-guided therapy as shown in prior randomized clinical trials [62]. The device allows the clinician to remotely monitor pulmonary artery pressures, continuously allowing for precise titration of medical therapies. Pulmonary artery pressures often become abnormal before clinical symptomology develops in patients with chronic heart failure. Thus, risk of heart failure hospitalization reduction in those with the device is likely based on the adjustment of medical therapies before the development of symptomatic congestion.

The CardioMEMS device might have the potential to be utilized in LVAD patients to monitor and adjust device parameters in response to hemodynamic status [63]. This is of course not without considering important limitations. Right heart failure is one of the comorbidities in LVAD patients where the use of this CardioMEMS may be less helpful as concordant changes in central venous pressures and pulmonary artery pressures do not always occur. Furthermore, patients with significant secondary pulmonary hypertension may have decoupling between diastolic pulmonary artery pressure and pulmonary capillary wedge pressure, also limiting the usefulness of the sensor.

### 5.2. Remote Dielectric Sensing (ReDS)

ReDS is another promising tool to noninvasively estimate intra-thoracic fluid levels (Figure 6), which may correlate with intracardiac filling pressures [64]. ReDS employs low-power electromagnetic signals emitted between two sensors (one each on the anterior and posterior body surfaces) embedded in a wearable vest. ReDS has been shown to have high sensitivity and moderate specificity to estimate pulmonary congestion [65]. It may also be used to screen for pulmonary congestion at an early stage for outpatients. However, elevated ReDS value does not necessarily indicate pulmonary congestion, and more precise tests such as right heart catheterization should be considered to distinguish other potential confounders such as pleural fluid accumulation related to pneumonia. Thus far, the clinical implication of ReDS in LVAD patients remains unknown.

### 5.3. HeartWare LVAD Flow Slope

**Figure 7:**
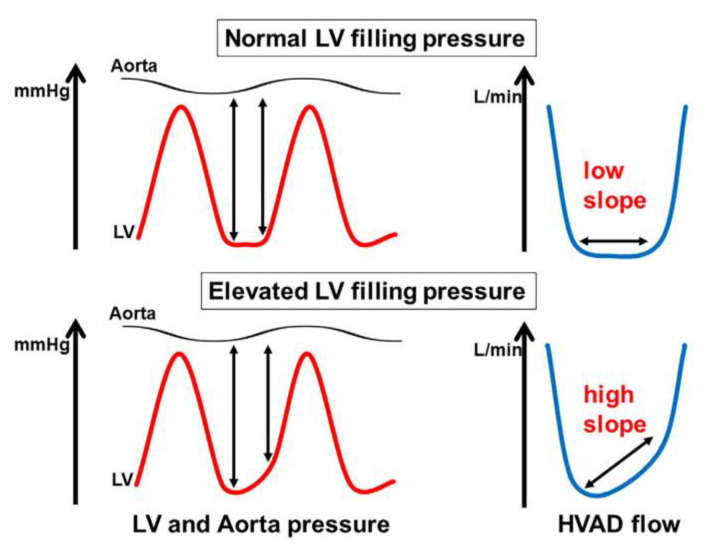
Association between left ventricular filling pressure and HeartWare LVAD flow slope [68].

The HeartWare LVAD provides an estimated instantaneous flow waveform that shows insights into patients and device properties. For example, low pulsatility and low mean flow indicate hypovolemia, whereas low pulsatility and high mean flow let us detect suspected device thrombosis. High pulsatility and low mean flow might indicate continuous suction, whereas high pulsatility and high mean flow indicate volume overload. LVAD flow is determined by the pressure difference between the aorta and left ventricle at a fixed device speed. When aortic pressure is assumed to be constant at the diastole phase, LVAD flow is dependent on left ventricular pressure. When left ventricular pressure increases, LVAD flow often increases. Given this mechanism, pulmonary capillary wedge pressure can be estimated by the slope of LVAD flow at the end-diastolic phase (Figure 7) [66]. We showed that the estimated elevated pulmonary capillary wedge pressure calculated from LVAD flow slope was associated with higher heart failure readmission rates [67].

Future advances in durable mechanical support may include a smart pump concept, which can automatically adjust device speed by continuously monitoring hemodynamic data points. For example, a novel smart pump might automatically measure the HeartWare LVAD flow slope and adjust its rotational speed considering estimated intra-cardiac pressure [66]. If the intra-cardiac pressure is estimated to be increased, the device speed would be increased to better unload the left ventricle.

## 6. Conclusions

Despite durable LVAD support, many patients can have abnormal hemodynamics due to a variety of clinical conditions even when clinically stable. LVAD therapy has improved survival in patients with advanced heart failure, though considerable limitations remain including unacceptably high readmission rates due to these various comorbidities, including volume overload and hemocompatibility-related adverse events. Assessment and optimization of hemodynamics might be one of the modifiable targets which could reduce the burden of these common post-implant complications. Interventions to optimize the hemodynamic status by adjustments of device speed and medications might improve clinical outcomes, though further large-scale prospective randomized control trials are needed to study these interventions. In the interim, noninvasive methods that estimate hemodynamics, including CardioMEMS, ReDS, and HartWare LVAD waveform analyses, may prove to be useful in more precisely guiding daily LVAD management.
